# Insights into the prognostic value and immunological role of CD74 in pan-cancer

**DOI:** 10.1007/s12672-024-01081-2

**Published:** 2024-06-11

**Authors:** Zebiao Liu, Mingquan Chen, Wanhua Zheng, Shicheng Yuan, Wenli Zhao

**Affiliations:** 1 Pathology, Huizhou First Hospital, Huizhou, 516000 China; 2 Guangxi Universities Key Laboratory of Stem cell and Biopharmaceutical Technology, School of Life Sciences, Guangxi Normal University, Guilin, 541004 China

**Keywords:** Pan-cancer, CD74, Immune microenvironment, Prognosis, Immunotherapy

## Abstract

**Background:**

CD74 is a non-polymorphic type II transmembrane glycoprotein. It is involved in the regulation of T and B cell development, and dendritic cell (DC) motility. Numerous studies have found that CD74 exerts an essential role in tumor immunity, but the expression profile of CD74 is still not systematically reported, and its value in human pan-cancer analysis is unknown. In this study, we analyzed the expression pattern of CD74 in 33 cancers, and evaluated the significance of CD74 in prognosis prediction and cancer immunity.

**Methods:**

Pan-cancer dataset from UCSC Xena.We used the Sangerbox website combined with R software’ Timer, CIBERSORT method and IOBR package to analyze and plot the data. Survival was assessed using the Kaplan—Meier method and log—rank test for 33 cancer types (p < 0.05). In addition, to explore the relationship between CD74 expression and immune checkpoints, immune cell infiltration, tumor mutational burden (TMB) and microsatellite instability (MSI), Spearman correlation analysis was performed.

**Results:**

This study comprehensively analyzed CD74 expression in 33 different tumor types, revealing that CD74 play an crucial role in cancer formation and development.

**Conclusions:**

CD74 gene expression in different cancers is associated with immune cell infiltration and immunomodulators and may provide a promising target for survival and immunotherapy. Our study shows that CD74 has an essential role as a biomarker of prognosis during tumor development, which highlights the possibility of new targeted therapies.

**Supplementary Information:**

The online version contains supplementary material available at 10.1007/s12672-024-01081-2.

## Introduction

Despite significant discoveries in cancer treatment in recent decades, cancer remains a global problem leading to millions of deaths [1–3]. In recent years, Cancer immunotherapy plays a central role in cancer treatment [4, 5]. Gene expression differences in various cancers have been detected with the innovation of sequencing. With the addition of sequencing tools and the continuous supplement of public databases, it is possible to explore new targets for immunotherapy by analyzing gene expression in a pan-cancer manner and evaluating its correlation with survival prognosis and immune cell infiltration. Aberrant expression of certain key genes across cancers may often be potential immunotherapeutic targets, and may be candidates for tumor immunotherapy targets if they reveal a correlation between them and immune cell infiltration, immune checkpoints, and that their aberrant expression affects certain immune-related signaling pathways.

The CD74, or HLA class II histocompatibility antigen γ chain was first discovered as the molecular chaperone of MHC class II [6], participating in the antigen presentation process mediated by MHC class II. And it is mainly highly expressed in some antigen-presenting immune cells [7], such as B cells, dendritic cells and macrophages. Nevertheless, under inflammatory conditions, CD74 will be upregulated in certain epithelial cells [8, 9]. The CD74 knockout mice confirmed that CD74 regulated mature B-cell survival through NF-κB-mediated transcriptional activation pathway [10].

Although the first discovered function of CD74 was as a chaperone for antigen-presenting molecules, subsequent research revealed further molecular interactions [11]. Macrophage migration inhibitory factor (MIF) is a natural ligand of CD74 [12], interestingly, both MIF and CD74 are linked to tumor development and metastasis: in metastatic melanoma [13], C36L1 peptide binds to CD74, thereby interfering with the MIF-CD74 signal transduction of dendritic cells and macrophages, and ultimately achieving the purpose of tumor treatment. Blocking MIF and CD74 signal transduction can inhibit tumor proliferation and increase tumor cell apoptosis in the development of hepatocellular carcinoma [14]. In a study of thyroid cancer, CD74 is overexpressed in tumor sites and its upregulation is associated with advanced tumor stage and poor prognosis [15]. What’s more, a recent study about hepatocellular carcinoma (HCC) had showed that CD74 was abundant in cancer tissues and mainly distributed on stromal macrophages. Blocking CD74 impaired the antitumor activity and proliferation of CD8 CTL in HCC [16]. CD74 expression is associations with immune cell infiltration and expression of programmed death ligand 1 (PD-L1) in the triple-negative breast cancer (TNBC) [17], moreover, CD74 promotes breast cancer cells metastasis, CD74 interacts with CD44 to promote the phosphorylation of CFL1 through RH0A-R0CK1 pathway, which in turn promotes tumor formation and metastasis, and this regulatory process may be independent of MIF [18]. It can predict the value of immune checkpoint therapy. There is growing evidence that CD74 may take an important part in human cancer.

Nevertheless, the majority of research on the role of CD74 in cancer are restricted to a specific tumor, and there are no pan-cancer studies about CD74 expression. Therefore, we used The Cancer Genome Atlas (TCGA) databases to evaluated the expression of CD74 in more than 30 different types of cancer, and we also evaluated their relationship with prog-nosis in different types of malignancy. This study provides a new perspective for the role of CD74 in tumor immunotherapy.

## Methods

### Data collection and mapping

The standardized pan-cancer datasets from the TCGA databases were downloaded using the interface at UCSC Xena (https://xenabrowser.net/). We plotted the full image of the article using Sangerbox (http://vip.sangerbox.com/) [19], a portal specializing in tumor bioinformatics analysis, who collects all the data from databases such as TCGA & GEO, which are widely used in clinical applications.

### Significance analysis of difference

We calculated the expression differences in the normal and tumor samples across tumors using Sangerbox and analyzed the differences using unpaired Wilcoxon rank sum test and signed rank test [20]. Gene expression level data in various cancer cell lines from CCLE database (https://portals.broadinstitute.org/ccle/). Real-Time PCR and Western blot further validates CD74 expression in colorectal cancer.

### Real-time PCR and western blot

We analyzed CD74 mRNA expression levels in colorectal cancer using real time PCR (RT-PCR) (SYBR Green Real-Time PCR Master Mixes and a Lightcycler System). Total RNA was isolated and 1 μg reverse transcribed with oligo dT primers using a first-strand cDNA synthesis kit (Promega, Madison, WI, USA) according to manufacturer’s instructions. Then, cDNAs were PCR amplified (40 cycles at 95 °C for 10 s, 60 °C for 30 s, and 72 °C for 30 s). Data were processed using the 2^−△△Ct^ method. The following primers were used: β-actin forward 5′-CTCCATCCTGGCCTCGCTGT-3′; β-actin reverse 5′-GCTGTCACCTTCACCGTTCC-3′; CD74 forward 5′-GACTCCGATCCAGTAGCCTAC-3′; CD74 reverse 5′-ACCGAGCCTCCGACAAGTGTAG-3. To examine the level of CD74 protein expression in colon cancer patients, cells were lysed in RIPA lysis buffer with protease inhibitor (1 mmol/L PMSF, Sigma). Cell debris was removed by centrifugation at 14 000 g at 4 °C for 30 min in an ice bath. Lysates were separated by 12% (w/v) SDS-PAGE. Proteins were then transferred to a nitrocellulose membrane and incubated with CD74 primary antibody (Cell Signaling) for 1 h before detection with horseradish peroxidase-conjugated secondary antibody (ThermoFisher).

### Immunohistochemistry (IHC) staining

To evaluate differences in CD74 expression at the protein level, IHC images of CD74 protein expression in normal tissues and five tumors tissues, including,were downloaded from the HPA (http://www.proteinatlas.org/) and analyzed.

### The relationship between CD74 in different tumors and prognosis and clinical phenotype

We investigated the connection of CD74 expression with prognosis based on the survival and clinical phenotype information of each tumor patient. Four key indicators were selected: overall survival (OS), disease-specific survival (DSS), disease-free interval (DFI), and progression-free interval (PFI). The Kaplan–Meier (KM) method and log-rank test were used to analyze the survival of each cancer type (p < 0.05). Survival curves were plotted using the R package ‘Survival’ and ‘Survminer’. In addition, Cox analysis was performed using the R package ‘survival’ and ‘forest plot’ to determine the connection of CD74 expression with survival in a variety of cancers [21].

### Correlation analysis of CD74 expression with immune cell infiltration across cancers

Based on the expression of CD74 in different tumors, the stromal, immune scores were calculated for each patient in each tumor using the ESTIMATE package in R [22]. We used the CIBERSORT method of IOBR in the R package to score immune cells in pan-cancer and perform a spearman correlation analysis with CD74 expression [23]. We also re-evaluated the MHC, EC, SC, CP, AZ, IPS infiltration scores for each patient in each tumor using the deconvo_ips method of the R package IOBR.

#### Correlation analysis of CD74 expression with TMB and MSI in tumors

In recent years, there are increasing reports on TMB, which is highly correlated with the efficacy of PD-1/PD-L1 inhibitors. Some tumor patients can predict the efficacy of immunotherapy by TMB markers. MSI is caused by defective DNA mismatch repair function in tumor tissues. MSI with defective DNA mismatch repair has emerged as an important clinical tumor marker. The TMB of tumors was calculated by the TMB function of the R package maftoolsM. To examine the correlation of CD74 expression in different tumors with TMB and MSI, log2(x + 0.001) transformation was first performed for each expression value, followed by spearman correlation analysis.

#### Gene set enrichment analysis

Using JAVA (http://software.broadinstitute.org/gsea/index.jsp), we conducted GSEA to assess for possible underlying mechanisms based on the ‘Molecular Signatures Database’ of h.all.v2022.1.Hs.symbols, c5.go.v2022.1.Hs.symbols and c2.cp.kegg.v2022.1.Hs.symbols. We applied R software (http://r-project.org/) and Bioconductor (http://bioconductor.org/) to visualize our results. Here we take LIHC and STAD as examples.

### Declarations

Our study involves human tissues derived from tumor tissues and normal tissues of colorectal patients. All patients signed an informed consent form, and the Clinical Trial Ethics Committee of the Huizhou First Hospital has approved the study. All experiments were performed in accordance with the relevant guide-lines and regulations of the Clinical Trial Ethics Committee of the Huizhou First Hospital.

#### Statistical analysis

CD74 expression data were normalized by log2(x + 0.001) transformation and further analyzed. Correlation studies were performed with spearman correlation analysis.

## Results

### Differential expression of CD74

**Fig. 1:**
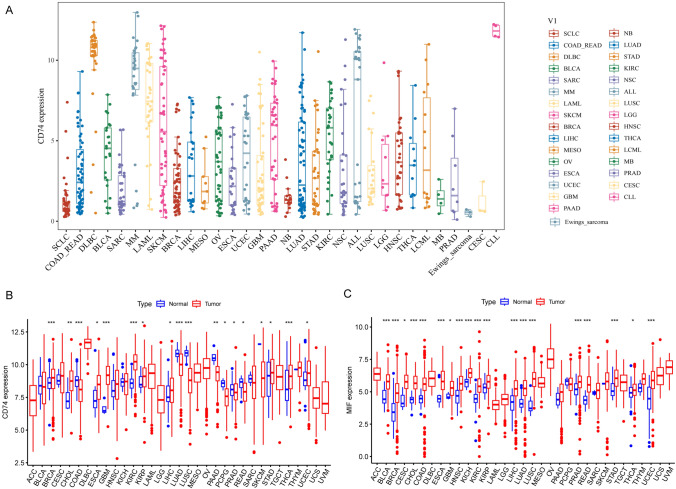
Pan-cancer CD74 and MIF expression. **A** The mRNA level of CD74 in CCLE. **B** The expression of CD74 between tumor tissues and normal tissues in TCGA database. **C** The expression of MIF between tumor tissues and normal tissues. *P < 0.05, **P < 0.01, ***P < 0.001

According to CCLE analysis, the gene expression levels of CD74 are different in various cancer cell lines (Fig. 1A). The results downloaded from UCSC displayed that CD74 was expressed in all cancres and up-regulated in breast invasive carcinoma (BRCA), cholangiocarcinoma (CHOL), glioblastoma multiforme (GBM), esophageal carcinoma (ESCA), kidney renal clear cell carcinoma (KIRC), kidney renal papillary cell carcinoma (KIRP), liver hepatocellular carcinoma (LIHC), stomach adenocarcinoma (STAD), thyroid carcinoma (THCA) and uterine corpus endometrial carcinoma (UCEC) (Fig. 1B). It was significantly downregulated in colon adenocarcinoma (COAD), lung squamous cell carcinoma (LUSC), lung adenocarcinoma (LUAD), pancreatic adenocarcinomaancreatic adenocarcinoma (PAAD), pheochromocytoma and paraganglioma (PCPG), prostate adenocarcinoma (PRAD), rectal adenocarcinoma (READ), skin cutaneous melanoma (SKCM). Since cell surface CD74 acts as a receptor for the cytokine macrophage migration inhibitory factor (MIF), and their combination induces a signaling cascade that regulates cell proliferation and survival, we also compared the expression of MIF in pan-cancer. MIF expression levels were remarkably elevated in 19 cancer types in pan-cancer (Fig. 1C).

Subsequently, we compared the IHC results provided by the HPA database with the CD74 gene expression data from TCGA (Supplementary Fig. 1), and found that the CD74 expression results were consistent.

### Expression of CD74 in colorectal cancer

**Fig. 2:**
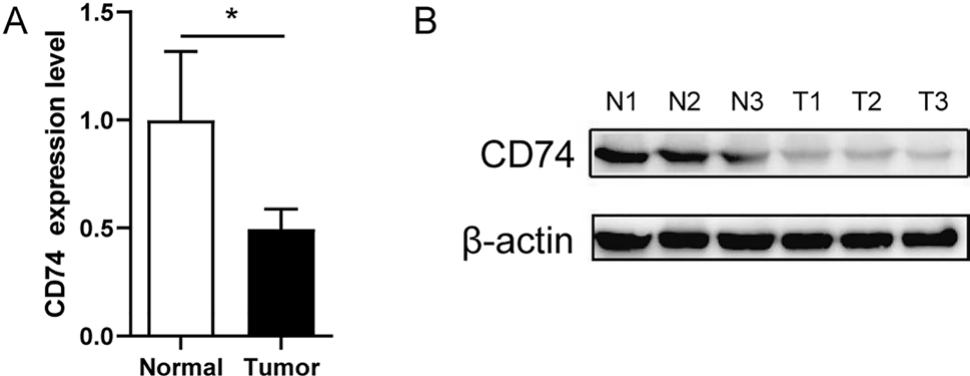
CD74 expression in colorectal cancer. **A** The mRNA relative expression level of CD74 in colorectal cancer. **B** Expression of CD74 protein levels. N1-3 is normal tissue, T1-3 is tumor tissue

We further validated CD74 expression in colorectal cancer using Real-Time PCR and Western blot. The results were consistent with the above (Fig. 2A, B). Both qPCR and WB results showed decreased expression of CD74 in colorectal cancer*.*

### Multiple prognostic value analysis of CD74

**Fig. 3:**
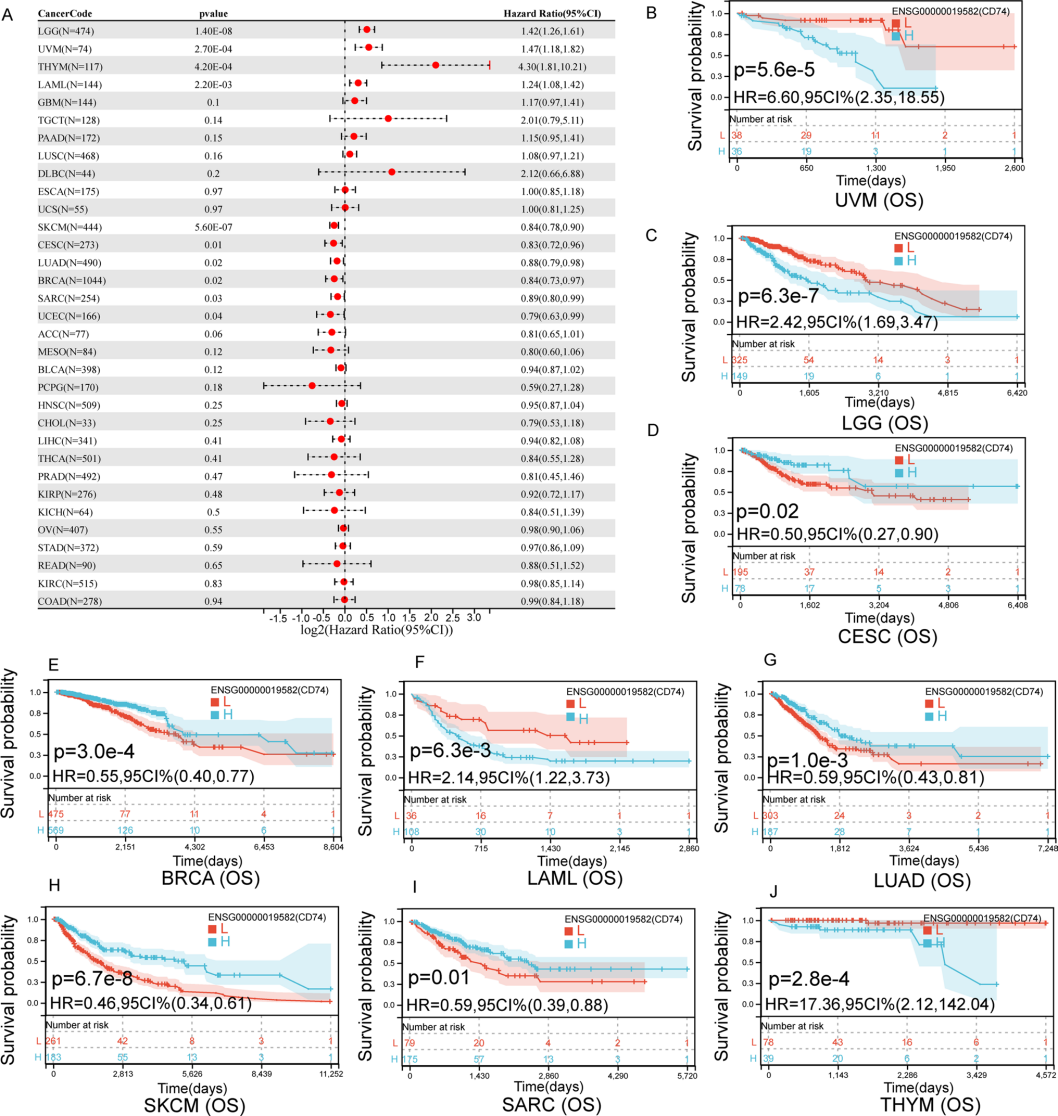
The relationship between the level of CD74 and OS in tumor patients. **A** OS related forest map. **B**–**J** Kaplan—Meier survival curves in UVM, LGG, CESC, BRCA, LAML, LUAD, SKCM, SARC and THYM

We have statistically examined the expression of CD74 in the whole cancer dataset and correlated it with patient prognosis. Survival indicators include DFI, PFI, DSS and OS. Cox regression analysis showed that CD74 expression was remarkably linked to OS in 10 cases of cancer (Fig. 3A), including LGG, UVM, THYM, LAML, SKCM, CESC, LUAD, BRCA, SARC and UCEC. Further, CD74 was a high-risk gene in LGG (hazard ratio = 1.42), UVM (hazard ratio = 1.47), THYM (hazard ratio = 4.3), LAML (hazard ratio = 1.24). To investigate the effect of CD74 expression expression on OS, we performed a KM analysis, which showed that in patients with higher CD74 expression in UVM (Fig. 3B), LGG (Fig. 3C), LAML (Fig. 3F) and THYM (Fig. 3J), the 5 year survival rate was lower.In CESC (Fig. 3D), BRCA (Fig. 3E), LUAD (Fig. 3G), SARC (Fig. 3I) and SKCM (Fig. 3H), higher levels of CD74 in patients were associated with better OS.

**Fig. 4:**
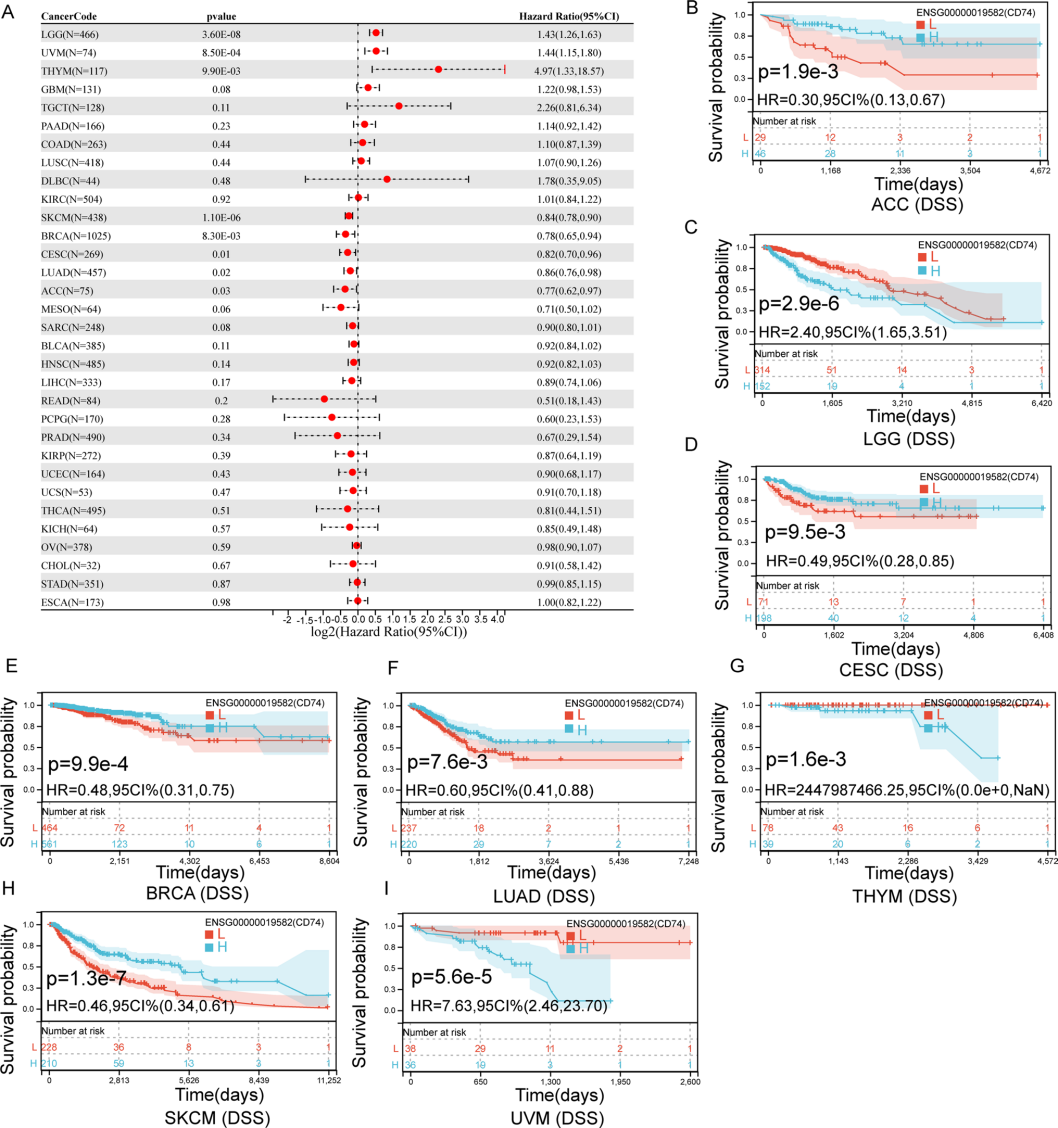
The relationship between the level of CD74 and DSS in tumor patients. **A** DSS related forest map. **B**–**I** Kaplan—Meier survival curves in ACC, LGG, CESC, BRCA, LUAD, THYM, SKCM and UVM

We also evaluated the association between CD74 levels in all tumors and DSS in cancer patients, and found that CD74 expression affected DSS in LGG, UVM, THYM, SKCM, BRCA, CESC, LUAD and ACC (Fig. 4A). In LGG (Fig. 4C), THYM (Fig. 4G) and UVM (Fig. 4I), higher levels of CD74 in patients were associated with poorer DSS. In CESC (Fig. 4D), BRCA (Fig. 4E), LUAD (Fig. 4F), SKCM (Fig. 4H) and ACC (Fig. 4B), higher levels of CD74 in patients were associated with better DSS.

**Fig. 5:**
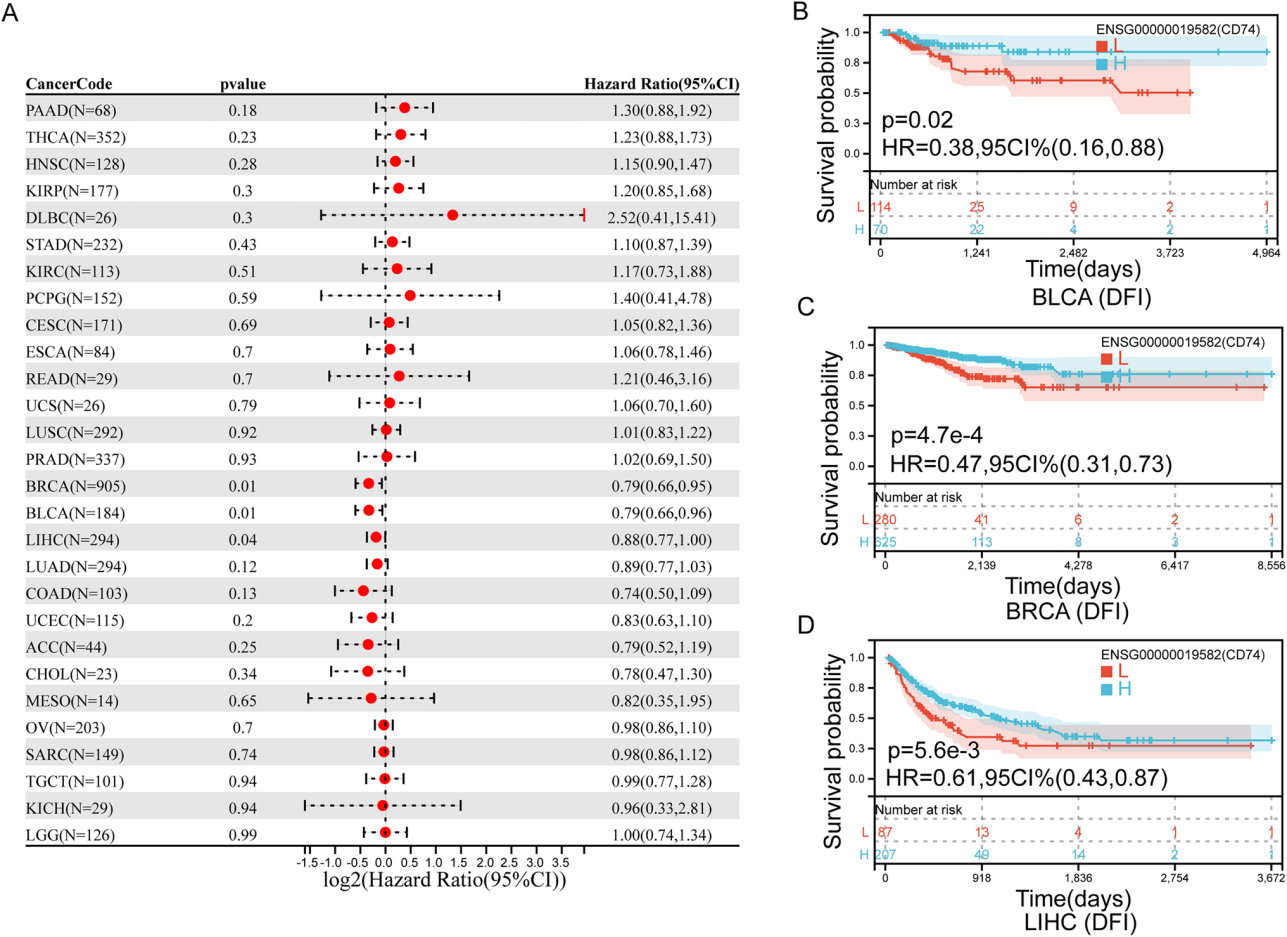
Association between the CD74 expression and the DFI of cancer patients. **A** Forest plot of DFI associations in 33 types of tumor. **B**–**D** Kaplan–Meier survival curves of DFI for patients stratified by the different expressions of CD74 BLCA, BRCA and LIHC

In addition, we found that the expression of CD74 affected the DFI of three cancers (Fig. 5A), including BLCA, LIHC and BRCA. In BLCA (Fig. 5B), BRCA (Fig. 5C) and LIHC (Fig. 5D), higher levels of CD74 in patients were associated with better DFI.

**Fig. 6:**
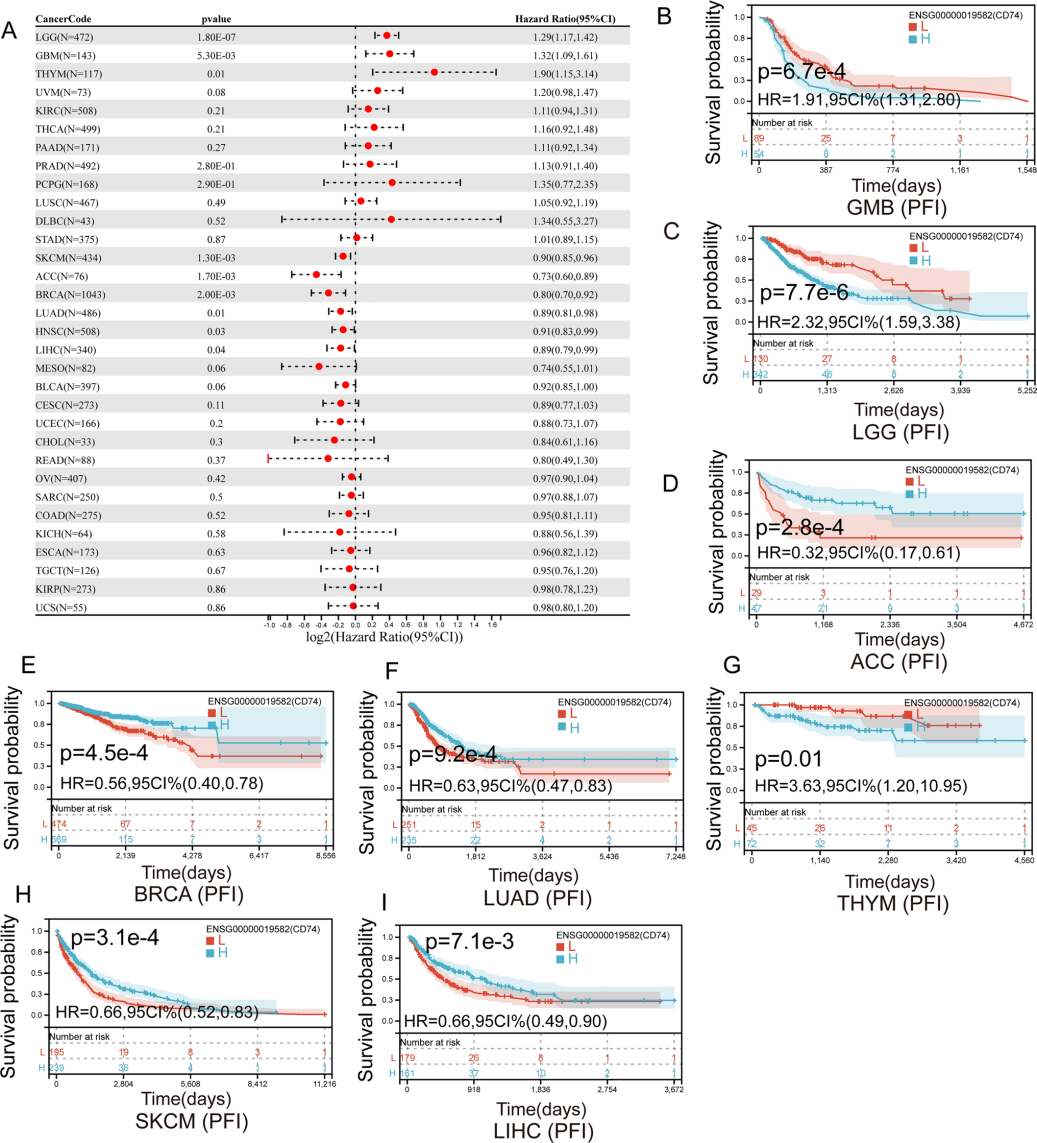
Association between the CD74 expression and the PFI of cancer patients. **A** Forest plot of PFI associations in 33 types of tumor. **B**–**I** Kaplan–Meier survival curves of PFI for patients stratified by the different expressions of CD74 GMB, LGG, ACC, BRCA, LUAD, THYM, SKCM, LIHC

We also found that the expression of CD74 affected the PFI of 11 cancers (Fig. 6A), including LGG, GMB, THYM, PRAD, PCPG, SKCM, ACC, BRCA, LUAD, HNSC and LIHC. In GMB (Fig. 6B), LGG (Fig. 6C) and THYM (Fig. 6G), higher levels of CD74 in patients were associated with poorer PFI. Patients with increased CD74 levels showed superior PFI to those with decreased CD74 levels in ACC (Fig. 6D), BRCA (Fig. 6E), LUAD (Fig. 6F), SKCM (Fig. 6H) and LIHC (Fig. 6I).

### Correlation analysis of CD74 expression and immune cell infiltration in pan-cancer

**Fig. 7:**
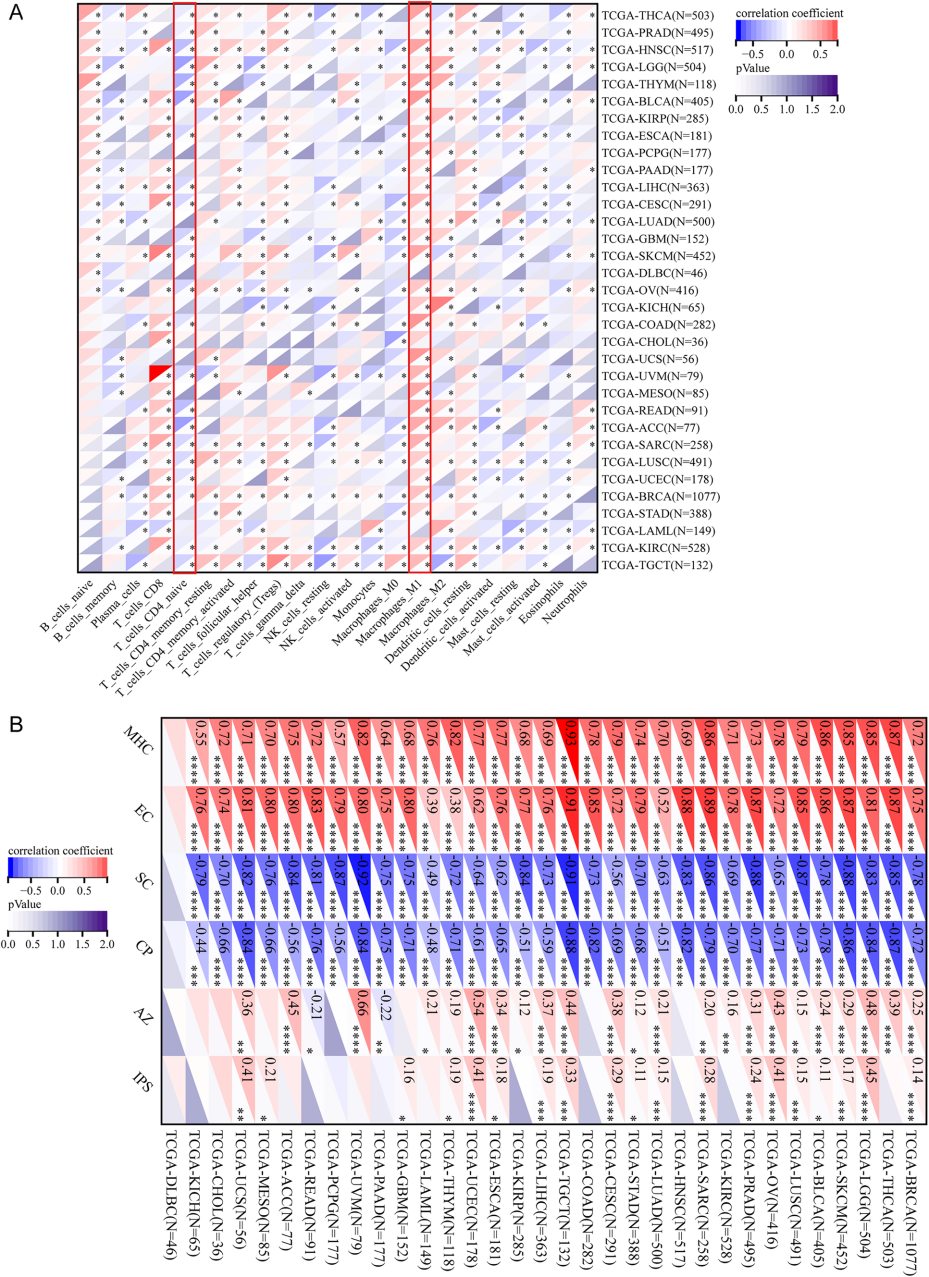
Immune cell infiltration analysis. **A** CIBERSORT method was used to compare the relationship between CD74 expression and tumor invasion of different immune cells. **B** MHC, EC, SC, CP, AZ, IPS infiltration score of each tumor was assessed by deconvo_ips method. The asterisks indicate a statistically significant p-value calculated using spearman correlation analysis. *P < 0.05

To clarify the association of CD74 with immune response, we analyzed the relationship between CD74 expression in each tumor and infiltration of immune cells using the Timer method of the IOBR package in R software. CD74 expression was significantly correlated with infiltration of immune cells. Importantly, CD74 expression was significantly positively correlated with M1 macrophages in all cancers except DLBC, LAML, and CHOL (Fig. 7A and Supplementary Fig. 2). CD74 expression was significantly negatively correlated with native CD4 T cell in all tumors except LAML, STAD, MESO, UCS, CHOL, DLBC, KICH, LUAD, PCPG, PAAD and KIRP. The results of MHC, EC, SC, CP, AZ, and IPS infiltration scores for each tumor showed that CD74 expression in most tumors was positively correlated with MHC, EC, and negatively correlated with SC, CP (Fig. 7B). This suggests that our abnormal expression of CD74 can affect numerous immune responses.

#### Correlation analysis with immunescore

**Fig. 8:**
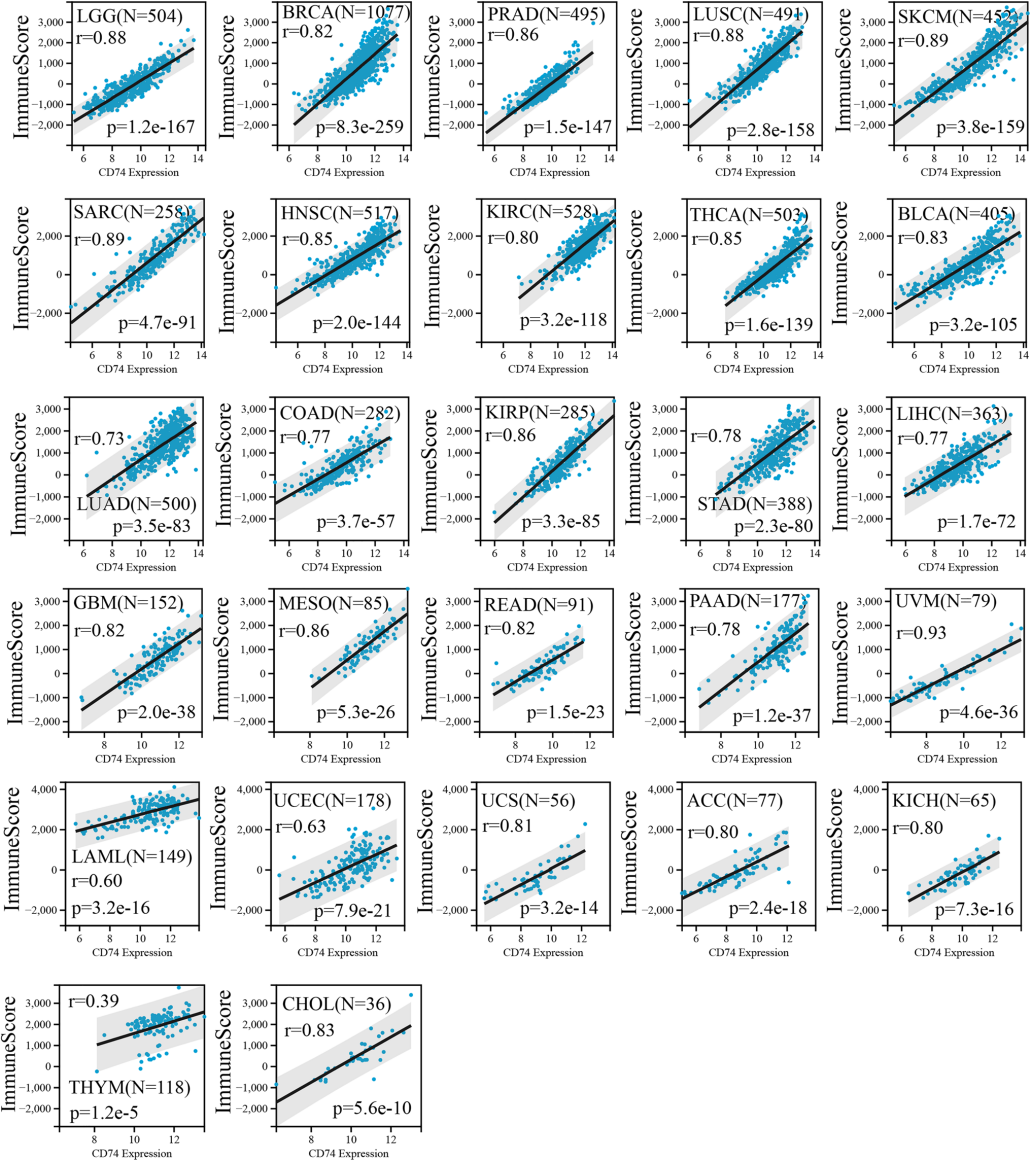
The relationship between CD74 expression and ImmuneScore. The results showed that CD74 expression in most tumors was significantly and positively correlated with ImmuneScore

We further analyzed the relationship between the expression of CD74 and ImmuneScore, and StromalScore. The results showed that CD74 expression in most tumors was significantly and positively correlated with ImmuneScore (Fig. 8), StromalScore (Supplementary Fig. 3).

### Relationship between CD74 expression and immunomodulators

**Fig. 9:**
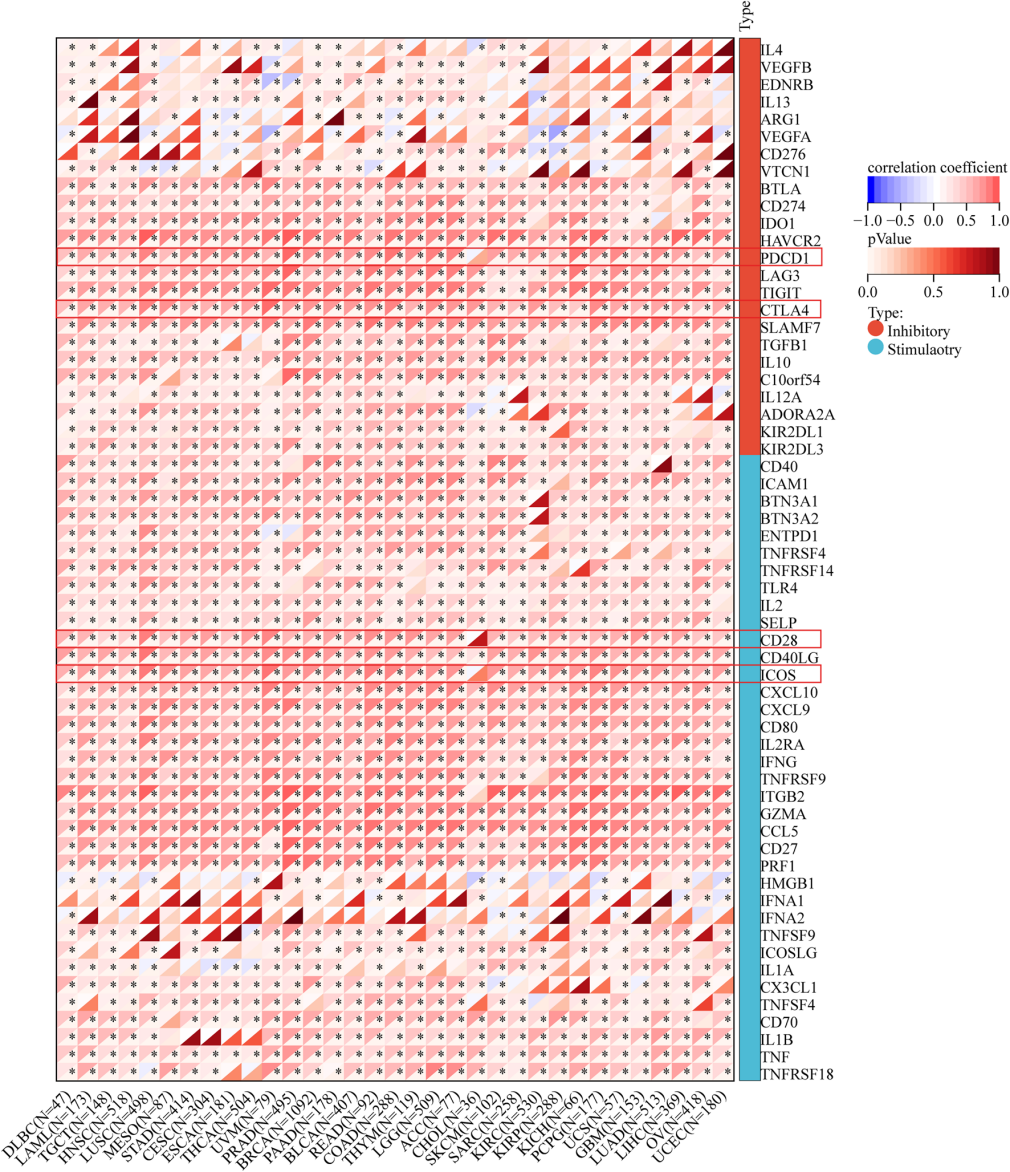
Correlation between CD74 and 60 immunomodulators (Inhibitory(24)、Stimulatory(36)). The asterisks indicate a statistically significant p-value calculated using spearman correlation analysis. The upper left corner of each square represents the correlation coefficient by color change, and the lower right corner is the p-value by color change; p < 0.05 indicates statistical significance. *P < 0.05

Immune checkpoints can affect the prognosis of cancer patients. Tumors evade immune responses through immune checkpoints such as PD-1 and PD-L1 [24]. We evaluated the correlation of CD74 expression with immunomodulators to clarify the immune function of CD74, which is essential to identify the tumor types for CD74 immunotherapy. The results of the study showed that CD74 expression in pan-cancer was positively correlated with most immune checkpoints (Fig. 9). Specifically, all tumors were positively correlated with CTLA4. All prognosis-related cancers except LAML were positively correlated with 6 immune checkpoints of CD27, TNF, CXCL9, CD40LG, TIGIT and SELP. All prognosis-related cancers except LAML and THYM were positively correlated with 3 immune checkpoints of ICOS, CD28 and PDCD1.

### Correlation of CD74 expression with TMB and MSI

**Fig. 10:**
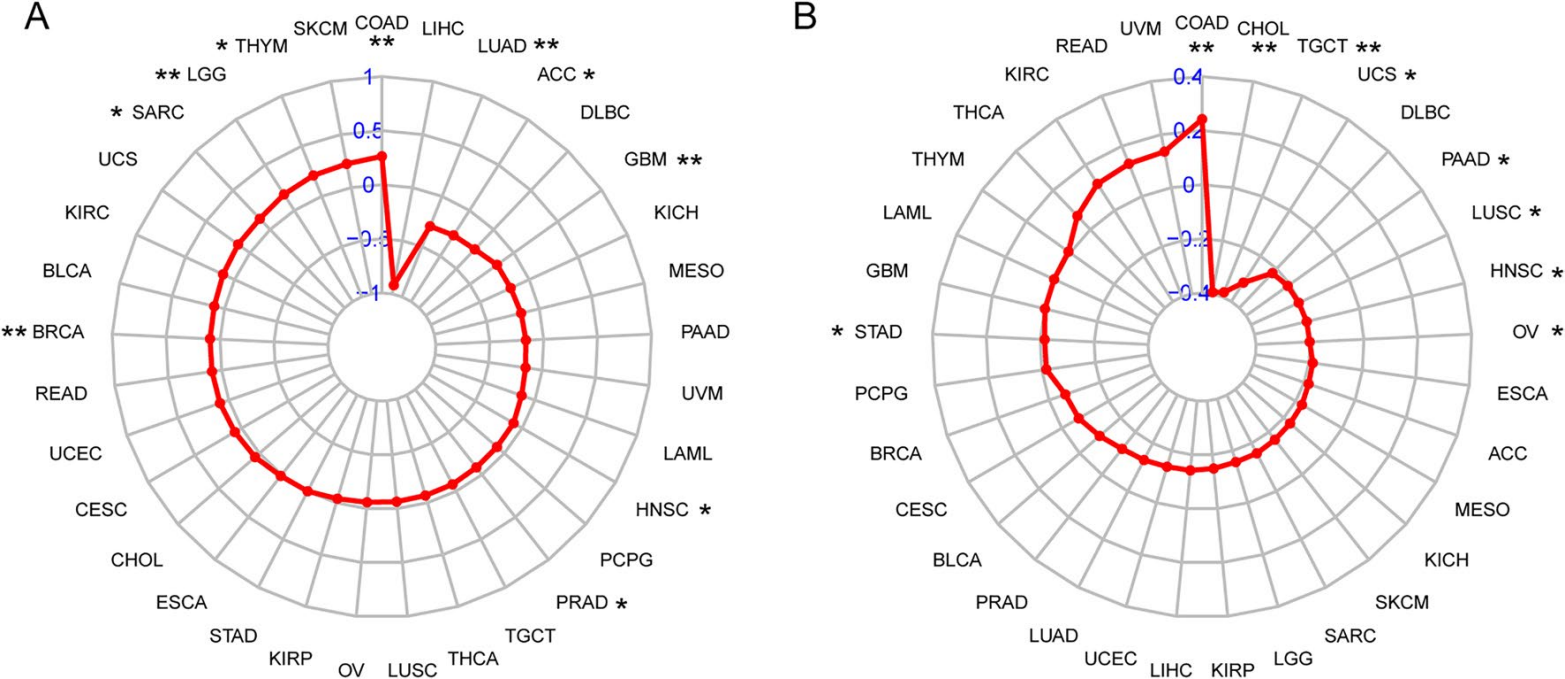
Associations between CD74 expression and tumor mutational burden (TMB) and microsatellite instability (MSI). **A** The association between CD74 expression and TMB levels in tumors. **B** The association between CD74 expression and MSI event in tumors

We also investigated the relationship between CD74 and TMB, MSI, two new biomarkers relevant to immunotherapy. We found that CD74 expression was positively correlated with TMB expression in many tumors, such as COAD and THYM. In addition, LUAD and ACC were negatively correlated with it (Fig. 10A). Finally, the findings demonstrated that CD74 expression was correlated with MSI, indicating that high CD74 expression in TGCT, UCS, CHOL, PAAD, HNSC, LUSC and OV was negatively correlated with MSI and positively correlated in COAD (Fig. 10B).

### GSEA of CD74 analysis

**Fig. 11:**
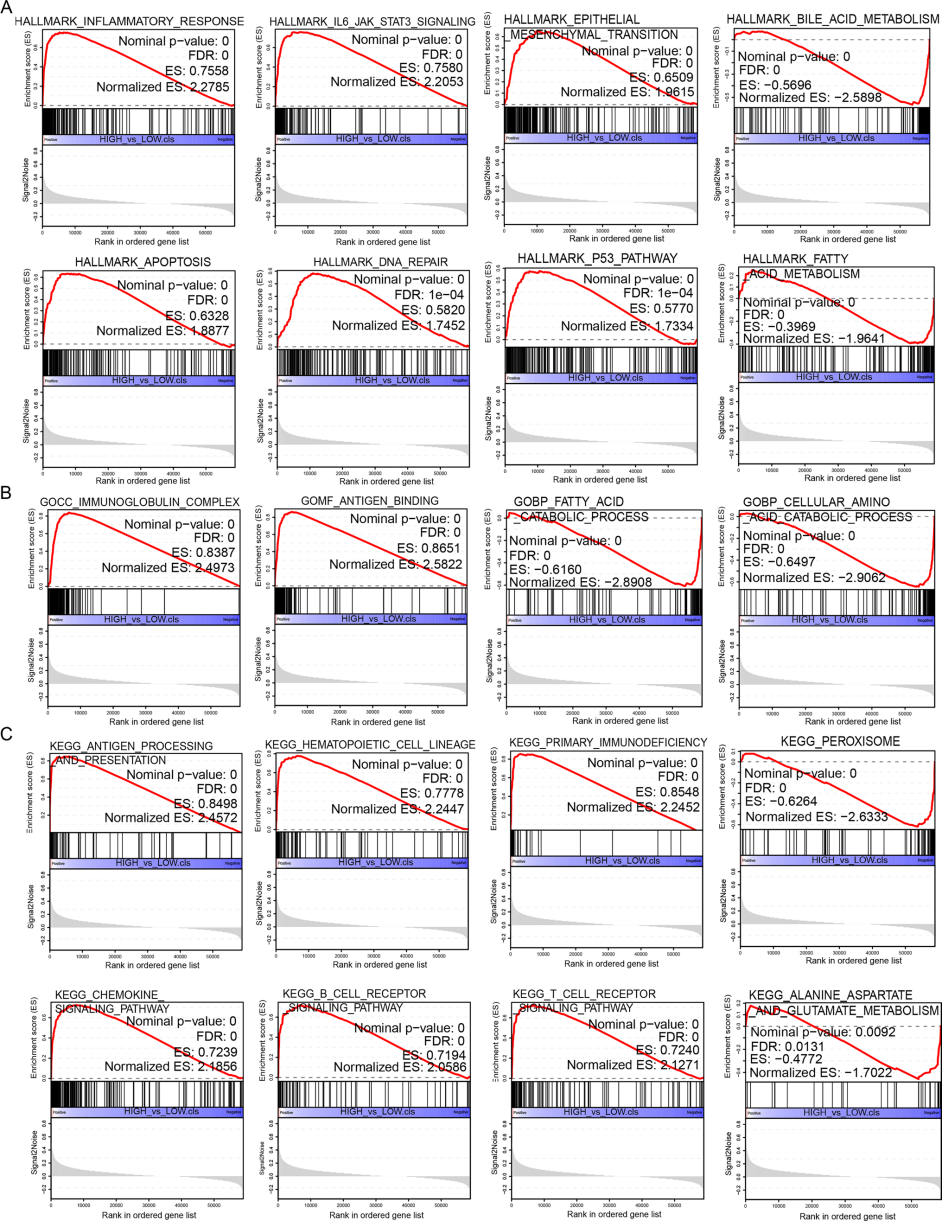
Analysis results of GSEA. **A** HALLMARK analysis of CD74 in LIHC. **B** GO functional annotation of CD74 in LIHC. **C** KEGG pathway analysis of CD74 in LIHC. The chart above shows only some of the results. Peaks on the upward curve indicate positive regulation and peaks on the downward curve indicate negative regulation. Here we show only a few of these signaling pathways

To investigate the biological function of CD74 expression in different tumor tissues, we evaluated the pathway through which CD74 may involve using GSEA. The results showed that in LIHC, CD74 was significantly associated with IL6/JAK2/STAT3 signaling pathway, epithelial mesenchymal transition, apoptosis, DNA repair, P53 signaling pathway, antigen presentation, chemokine signaling pathway, and B and T cell receptor signaling pathway (Fig. 11A–C). It is important to note that many of the same pathways can be found in the GSEA analysis of STAD. It gates mostly associated with immune signaling pathway, inflammatory factor related pathways, P53 signaling pathway, epithelial-to-mesenchymal transition (Supplementary Fig. 4). These remarkable pathways may be the key pathways underlying the involvement of CD74 in tumorigenesis and development.

## Discussion

With the progress of human society and the improvement of material living standards, cancer is increasingly becoming a major obstacle to people's well-being. In recent years, scientists have proposed that to combat the intricacies of tumors, clinical trials should consider combining gene focused therapies with immune-tailored approaches (e.g., checkpoint resistance, chimeric antigen receptor T cells, etc.), hormonal therapies, chemotherapy, and/or new drugs [25]. The latest treatments for cancer involve surgical resection, chemotherapy and radiation therapy, but their efficacy is limited and they are not effective in terminal cancer [26]. Early screening and precise treatment of cancer is an effective tool for cancer prevention and treatment. Pan-cancer analysis can establish links between different cancers, and can identify similarities from different cancers as well as differences from the same place. This provides a broader and effective idea for cancer treatment. In recent years, there has been an increasing number of studies on immune checkpoint blockade therapies such as PD-1, PD-L1, and CTLA4, which, combined with interventions in immune-related pathways, can effectively improve the clinical manifestations of cancer [6, 27]. Our results also show that CD74 expression in most tumors is positively correlated with CTLA4 and PD1 (Fig. 9), and it is worthwhile to further investigate whether CD74 can be used as a key therapeutic target in clinical studies. A recent study on CD74 and CTLA-4 is consistent with our results, and they also indicate that the gene expression level of CD74 serves as a potential predictor of anti-PD-1/CTLA-4 immunotherapy in a wide range of cancer types, which further emphasizes the potential predictive value of CD74 in tumor [28].

In this study, we first determined the expression of CD74 in pan-cancer. We performed a comprehensive assessment of CD74 expression in different cancers using TCGA, and the results were consistent with previous studies [15, 16]. The findings showed that CD74 expression was significantly enhanced in 10 cancers and significantly decreased in 8 cancers (Fig. 1A). MIF expression was significantly enhanced in 19 cancer types in pan-cancer (Fig. 1B). The effect of the joint action between MIF and CD74 on malignant tumors has not been systematically reported, and the asynchrony of their abnormal expression deserves further investigation.

Differential expression of CD74 was associated with worse DSS, DFI, OS or PFI in a few cancers (Figs. 3, 4, 5, 6). Kaplan–Meier viability profile demonstrates that high expression of CD74 is notably linked to poor OS in LGG, CESC, BRCA, LAML, LUAD, SARC, THYM, SKCM and UVM (Fig. 3B–J). CD74 was a protecting element for CESC, LUAD, BRCA, SARC and SKCM, and a danger element for LAML, THYM, LGG, and UVM. For the DSS, one-way Cox regression analysis showed that CD74 was a protecting element for sufferers of CESC, LUAD, BRCA, SKCM and ACC, and a hazard element for sufferers of LGG, THYM and UVM (Fig. 4). However, the viability of clinical studies may be reduced by using OS as an endpoint, and mortality from non-cancerous reasons does not consistently reveal tumor biology, aggressiveness, or response to therapeutic Therefore, we further analyzed DFI and PFI. For DFI, the findings of one-way Cox regression analysis demonstrated that CD74 was protective for sufferers with BLCA, BRCA, LIHC (Fig. 5). Finally, PFI results showed that CD74 could be a protective factor in LUAD, ACC, BRCA, LIHC, SKCM and a hazard element for sufferers with GMB, LGG, THYM (Fig. 6). These results showed that CD74 played different roles in different tumors and should be targeted for clinical treatment.

To investigate the feasibility of CD74 and its antibodies as tumor autoimmunotherapy candidates, we explored the correspondence of CD74 expression in different tumors with immune cell infiltration and immune modulators. We found that CD74 expression in almost all tumors was positively correlated with infiltration of M1 macrophages (Fig. 7). Macrophages are a key factor in promoting tumor inflammation. Tumor-associated macrophages can have a yin and yang effect on the effectiveness of cytoreductive therapies (chemotherapy and radiotherapy), counteracting antitumor activity by coordinating pro-tumor, tissue repair responses, or conversely, contributing to their ultimate antitumor efficacy [29]. Inflammatory cells are an important component of the tumor ecology [30–33]. M1 macrophages act as inflammatory cells producing TNF-α, IL-1β and IL-12, creating an inflammatory environment that increases the risk of cancer [29, 34, 35]. A recent study has shown that CD74 promotes the activation and accumulation of Tregs in tumors [36]. Tregs are a major barrier to tumor immune rejection because they induce tumor immune tolerance and promote tumor angiogenesis [37]. Selective depletion or in-hibition of Tregs in tumors and preservation of Tregs in healthy tissue should eliminate tumors, especially when combined with other immunotherapies [36]. Our results showed that the expression of CD74 in KIRC and THCA was positively correlated with Tregs (Fig. 7A), in addition, the expression level of CD74 was significantly elevated in KIRC and THCA (Fig. 1B), which made us question whether inhibiting the expression of CD74 could effectively reduce tumor immune rejection, which needs to be verified by further experiments, and implied to us that CD74 could be a potential therapeutic target for KIRC and THCA.

Gene mutations are the primary cause of cancer formation [38]. Immune checkpoints are molecules that regulate T cells and can contribute directly to the suppression and stimulation of immune cell function [39–41]. MSI is an essential biomarker in immune checkpoint suppressor [42]. Mutations occur throughout our lives. Small mutations have no significant impact on our normal life, and only when accumulated to a certain extent can they induce cancer. Our results showed that CD74 expression was critical linked to TMB, MSI (Fig. 10), suggesting that we can interfere with the level of CD74 to affect the occurrence of precancerous mutations in cancer.

Several clinical trials have been conducted with an anti-CD74 biologic (Milatuzumab), an immunotherapeutic drug targeting CD74, which had no serious adverse effects in patients with multiple myeloma and stabilized the disease for up to 12 weeks in some patients [43–45]. Studies have also reported no serious adverse effects of milatuzumab in humans in chronic lymphocytic leukemia, non-Hodgkin’s lymphoma, and multiple myeloma. cD74 has a role as a co-signaling molecule and has been implicated in the proliferation and survival of malignant B-cells [46]. The anti-CD74 monoclonal antibody LL1 has been humanized (hLL1 milatuzumab or IMMU-115) and may provide the basis for a novel therapeutic approach to B-cell malignancies, particularly because the antibody rapidly internalizes CD74 + malignant cells [47, 48]. Our study highlights the possibility of new targeted therapies. The non-negligible correlation of CD74 with a variety of immune cell infiltrates and immune checkpoints not only expands our understanding of CD74, but also provides additional entry points for CD74 to become a novel therapeutic target.

In conclusion, our first pan-cancer analysis of CD74 showed that CD74 expression in different cancers is associated with immune cell infiltration and immune checkpoints and that aberrant CD74 expression is associated with poor prognosis in multiple cancer types, with TMB and MSI and cancer immunity. It is an important candidate for survival and immunotherapy. Despite these conclusions from our data analysis, more plausible experiments are needed in the future to demonstrate a specific role in malignancy, which is the focus of our next research.

### Supplementary Information

Supplementary file 1 (DOCX 2934 KB)

## Data Availability

All standardized pan-cancer datasets were downloaded from the UCSC Xena (https://xenabrowser.net/) website. All data are from different biological samples and do not take into account for repeated tests.

## Abbreviations

KIRC: Kidney renal clear cell carcinoma
LIHC: Liver hepatocellular carcinoma
SKCM: Skin cutaneous melanoma
SARC: Sarcoma
THYM: Thymoma
THCA: Thyroid carcinoma
OV: Ovarian serous cystadenocarcinoma
PAAD: Pancreatic adenocarcinomaancreatic adenocarcinoma
TGCT: Testicular germ cell tumors
LAML: Acute myeloid leukemia
CHOL: Cholangiocarcinoma
GBM: Glioblastoma multiforme
LGG: Brain lower grade glioma
BRCA: Breast invasive carcinoma
CESC: Cervical squamous cell carcinoma and endocervical adenocarcinoma
ESCA: Esophageal carcinoma
KIRP: Kidney renal papillary cell carcinoma
COAD: Colon adenocarcinoma
STAD: Stomach adenocarcinoma
PRAD: Prostate adenocarcinoma
LUSC: Lung squamous cell carcinoma
READ: Rectal adenocarcinoma
ACC: Adrenocortical carcinoma
DLBC: Lymphoid neoplasm diffuse large B-cell lymphoma
PCPG: Pheochromocytoma and paraganglioma
BLCA: Bladder urothelial carcinoma
UCS: Uterine carcinosarcoma
KICH: Kidney chromophobe
UCEC: Uterine corpus endometrial carcinoma
LUAD: Lung adenocarcinoma
HNSC: Head and neck squamous cell carcinoma
MESO: Mesothelioma
UVM: Uveal melanoma
MIF: Macrophage migration inhibitory factor
TCGA: The Cancer Genome Atlas
OS: Overall survival
DSS: Disease-specific survival
DFI: Disease-free interval
PFI: Progression-free interval
KM: Kaplan–Meier

## References

[1] Kumari P, Dang S (2021). Anti-cancer potential of some commonly used drugs. Curr Pharm Des.

[2] Cheng X, Wang X, Nie K, Cheng L, Zhang Z, Hu Y, Peng W (2021). Systematic pan-cancer analysis identifies TREM2 as an immunological and prognostic biomarker. Front Immunol.

[3] Mun EJ, Babiker HM, Weinberg U, Kirson ED, Von Hoff DD (2018). Tumor-treating fields: a fourth modality in cancer treatment. Clin Cancer Res.

[4] Taefehshokr S, Parhizkar A, Hayati S, Mousapour M, Mahmoudpour A, Eleid L, Rahmanpour D, Fattahi S, Shabani H, Taefehshokr N (2022). Cancer immunotherapy: challenges and limitations. Pathol Res Pract.

[5] Jafarzadeh L, Khakpoor-Koosheh M, Mirzaei H, Mirzaei HR (2021). Biomarkers for predicting the outcome of various cancer immunotherapies. Crit Rev Oncol Hematol.

[6] Abril-Rodriguez G, Ribas A (2017). SnapShot: immune checkpoint inhibitors. Cancer Cell.

[7] Xu S, Li X, Tang L, Liu Z, Yang K, Cheng Q (2021). CD74 correlated with malignancies and immune microenvironment in gliomas. Front Mol Biosci.

[8] Su H, Na N, Zhang X, Zhao Y (2017). The biological function and significance of CD74 in immune diseases. Inflamm Res.

[9] Zhao S, Molina A, Yu A, Hanson J, Cheung H, Li X, Natkunam Y (2019). High frequency of CD74 expression in lymphomas: implications for targeted therapy using a novel anti-CD74-drug conjugate. J Pathol Clin Res.

[10] Lantner F, Starlets D, Gore Y, Flaishon L, Yamit-Hezi A, Dikstein R, Leng L, Bucala R, Machluf Y, Oren M (2007). CD74 induces TAp63 expression leading to B-cell survival. Blood.

[11] Schröder B (2016). The multifaceted roles of the invariant chain CD74–More than just a chaperone. Biochem Biophys Acta.

[12] Bucala R, Shachar I (2014). The integral role of CD74 in antigen presentation, MIF signal transduction, and B cell survival and homeostasis. Mini Rev Med Chem.

[13] Figueiredo CR, Azevedo RA, Mousdell S, Resende-Lara PT, Ireland L, Santos A, Girola N, Cunha R, Schmid MC, Polonelli L (2018). Blockade of MIF-CD74 signalling on macrophages and dendritic cells restores the antitumour immune response against metastatic melanoma. Front Immunol.

[14] Wirtz TH, Saal A, Bergmann I, Fischer P, Heinrichs D, Brandt EF, Koenen MT, Djudjaj S, Schneider KM, Boor P (2021). Macrophage migration inhibitory factor exerts pro-proliferative and anti-apoptotic effects via CD74 in murine hepatocellular carcinoma. Br J Pharmacol.

[15] Cheng SP, Liu CL, Chen MJ, Chien MN, Leung CH, Lin CH, Hsu YC, Lee JJ (2015). CD74 expression and its therapeutic potential in thyroid carcinoma. Endocr Relat Cancer.

[16] Xiao N, Li K, Zhu X, Xu B, Liu X, Lei M, Sun HC (2022). CD74(+) macrophages are associated with favorable prognosis and immune contexture in hepatocellular carcinoma. Cancer Immunol, Immunother: CII.

[17] Noer JB, Talman MM, Moreira JMA (2021). HLA class II histocompatibility antigen γ chain (CD74) expression is associated with immune cell infiltration and favorable outcome in breast cancer. Cancers.

[18] Liu Z, Chu S, Yao S, Li Y, Fan S, Sun X, Su L, Liu X (2016). CD74 interacts with CD44 and enhances tumorigenesis and metastasis via RHOA-mediated cofilin phosphorylation in human breast cancer cells. Oncotarget.

[19] Shen W, Song Z, Zhong X, Huang M, Shen D, Gao P, Qian X, Wang M, He X, Wang T (2022). Sangerbox: a comprehensive, interaction-friendly clinical bioinformatics analysis platform. iMeta.

[20] Liu J, Lichtenberg T, Hoadley KA, Poisson LM, Lazar AJ, Cherniack AD, Kovatich AJ, Benz CC, Levine DA, Lee AV (2018). An integrated TCGA pan-cancer clinical data resource to drive high-quality survival outcome analytics. Cell.

[21] Andersen PK, Gill RD (1982). Cox's regression model for counting processes: a large sample study. Ann Stat.

[22] Yoshihara K, Shahmoradgoli M, Martínez E, Vegesna R, Kim H, Torres-Garcia W, Treviño V, Shen H, Laird PW, Levine DA (2013). Inferring tumour purity and stromal and immune cell admixture from expression data. Nat Commun.

[23] Newman AM, Liu CL, Green MR, Gentles AJ, Feng W, Xu Y, Hoang CD, Diehn M, Alizadeh AA (2015). Robust enumeration of cell subsets from tissue expression profiles. Nat Methods.

[24] Mlecnik B, Bindea G, Pagès F, Galon J (2011). Tumor immunosurveillance in human cancers. Cancer Metastasis Rev.

[25] Tsimberidou AM, Fountzilas E, Nikanjam M, Kurzrock R (2020). Review of precision cancer medicine: evolution of the treatment paradigm. Cancer Treat Rev.

[26] Siegel RL, Miller KD, Jemal A (2019). Cancer statistics, 2019. CA Cancer J Clin.

[27] Deng R, Lu J, Liu X, Peng XH, Wang J, Li XP (2020). PD-L1 expression is highly associated with tumor-associated macrophage infiltration in nasopharyngeal carcinoma. Cancer Manag Res.

[28] Wang J, Li X, Xiao G, Desai J, Frentzas S, Wang ZM, Xia Y, Li B (2024). CD74 is associated with inflamed tumor immune microenvironment and predicts responsiveness to PD-1/CTLA-4 bispecific antibody in patients with solid tumors. Cancer Immunol, Immunother: CII.

[29] Zhou X, Du J, Liu C, Zeng H, Chen Y, Liu L, Wu D (2021). A pan-cancer analysis of CD161, a potential new immune checkpoint. Front Immunol.

[30] Mantovani A, Marchesi F, Malesci A, Laghi L, Allavena P (2017). Tumour-associated macrophages as treatment targets in oncology. Nat Rev Clin Oncol.

[31] Mantovani A, Allavena P, Sica A, Balkwill F (2008). Cancer-related inflammation. Nature.

[32] Diakos CI, Charles KA, McMillan DC, Clarke SJ (2014). Cancer-related inflammation and treatment effectiveness. Lancet Oncol.

[33] Hanahan D, Weinberg RA (2011). Hallmarks of cancer: the next generation. Cell.

[34] Shapouri-Moghaddam A, Mohammadian S, Vazini H, Taghadosi M, Esmaeili SA, Mardani F, Seifi B, Mohammadi A, Afshari JT, Sahebkar A (2018). Macrophage plasticity, polarization, and function in health and disease. J Cell Physiol.

[35] Wang S, Liu R, Yu Q, Dong L, Bi Y, Liu G (2019). Metabolic reprogramming of macrophages during infections and cancer. Cancer Lett.

[36] Bonnin E, Rodrigo Riestra M, Marziali F, Mena Osuna R, Denizeau J, Maurin M, Saez JJ, Jouve M, Bonté PE, Richer W (2024). CD74 supports accumulation and function of regulatory T cells in tumors. Nat Commun.

[37] Facciabene A, Motz GT, Coukos G (2012). T-regulatory cells: key players in tumor immune escape and angiogenesis. Can Res.

[38] Zhou L, Chong MM, Littman DR (2009). Plasticity of CD4+ T cell lineage differentiation. Immunity.

[39] Martincorena I, Campbell PJ (2015). Somatic mutation in cancer and normal cells. Science.

[40] Hu J, Xu J, Feng X, Li Y, Hua F, Xu G (2021). Differential expression of the TLR4 gene in pan-cancer and its related mechanism. Front Cell Dev Biol.

[41] Feng M, Jiang W, Kim BYS, Zhang CC, Fu YX, Weissman IL (2019). Phagocytosis checkpoints as new targets for cancer immunotherapy. Nat Rev Cancer.

[42] Lee DW, Han SW, Bae JM, Jang H, Han H, Kim H, Bang D, Jeong SY, Park KJ, Kang GH (2019). Tumor mutation burden and prognosis in patients with colorectal cancer treated with adjuvant fluoropyrimidine and oxaliplatin. Clin Cancer Res.

[43] Berkova Z, Tao RH, Samaniego F (2010). Milatuzumab—a promising new immunotherapeutic agent. Expert Opin Investig Drugs.

[44] Mark T, Martin P, Leonard JP, Niesvizky R (2009). Milatuzumab: a promising new agent for the treatment of lymphoid malignancies. Expert Opin Investig Drugs.

[45] Stein R, Qu Z, Cardillo TM, Chen S, Rosario A, Horak ID, Hansen HJ, Goldenberg DM (2004). Antiproliferative activity of a humanized anti-CD74 monoclonal antibody, hLL1, on B-cell malignancies. Blood.

[46] Stein R, Smith MR, Chen S, Zalath M, Goldenberg DM (2009). Combining milatuzumab with bortezomib, doxorubicin, or dexamethasone improves responses in multiple myeloma cell lines. Clin Cancer Res.

[47] Stein R, Mattes MJ, Cardillo TM, Hansen HJ, Chang CH, Burton J, Govindan S, Goldenberg DM (2007). CD74: a new candidate target for the immunotherapy of B-cell neoplasms. Clin Cancer Res.

[48] Martin P, Furman RR, Rutherford S, Ruan J, Ely S, Greenberg J, Coleman M, Goldsmith SJ, Leonard JP (2015). Phase I study of the anti-CD74 monoclonal antibody milatuzumab (hLL1) in patients with previously treated B-cell lymphomas. Leuk Lymphoma.

