# Supplementary material for: Insights into the prognostic value and immunological role of CD74 in pan-cancer
Source: Discov Oncol. 2024 Jun 11;15:222. doi: 10.1007/s12672-024-01081-2 (PMC11166624; doi:10.1007/s12672-024-01081-2)
Supplement: Supplementary file 1 — (DOCX 2934 KB) [file 12672_2024_1081_MOESM1_ESM.docx]

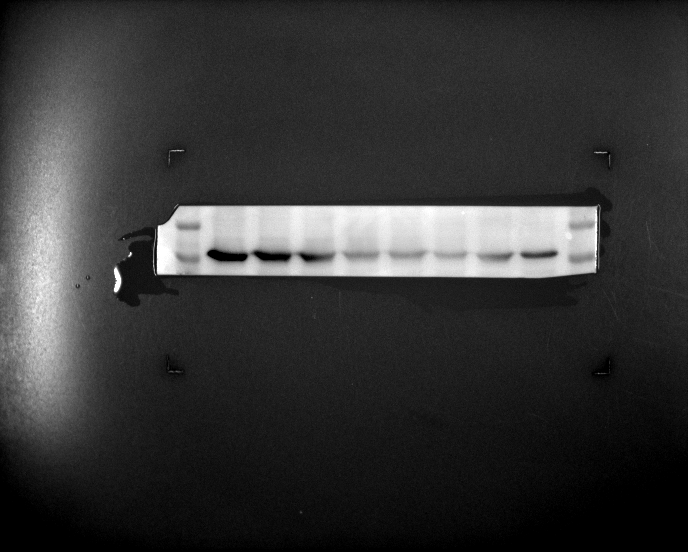


**CD74 expression in colon cancer, the three on the left are the normal group and the five on the right are the tumor group.**


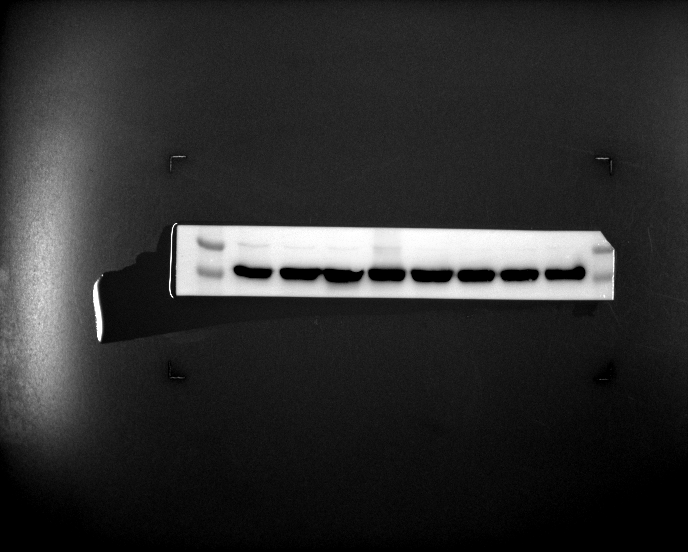


**β-actin. The left three were normal groups, and the right five were tumor groups.**


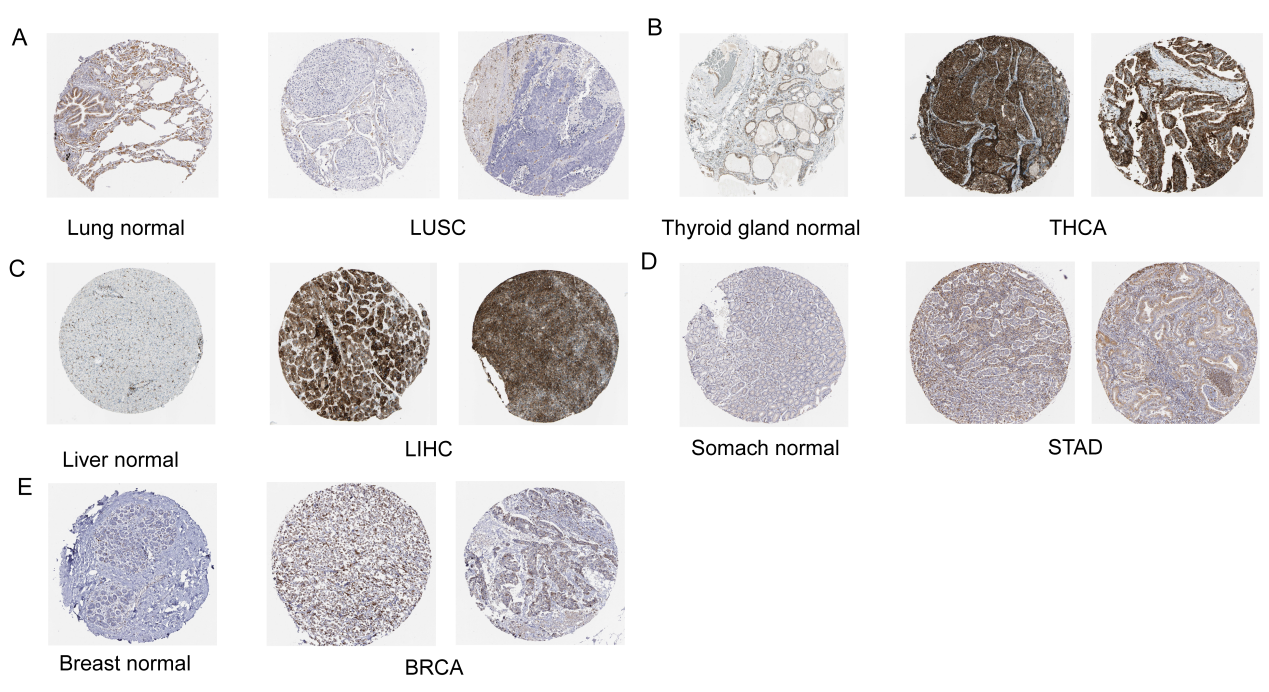


**Supplementary Figure 1. IHC results from the HPA database. (A) LUSC. (B) THCA. (C) LIHC. (D) STAD. (E) BRCA.**


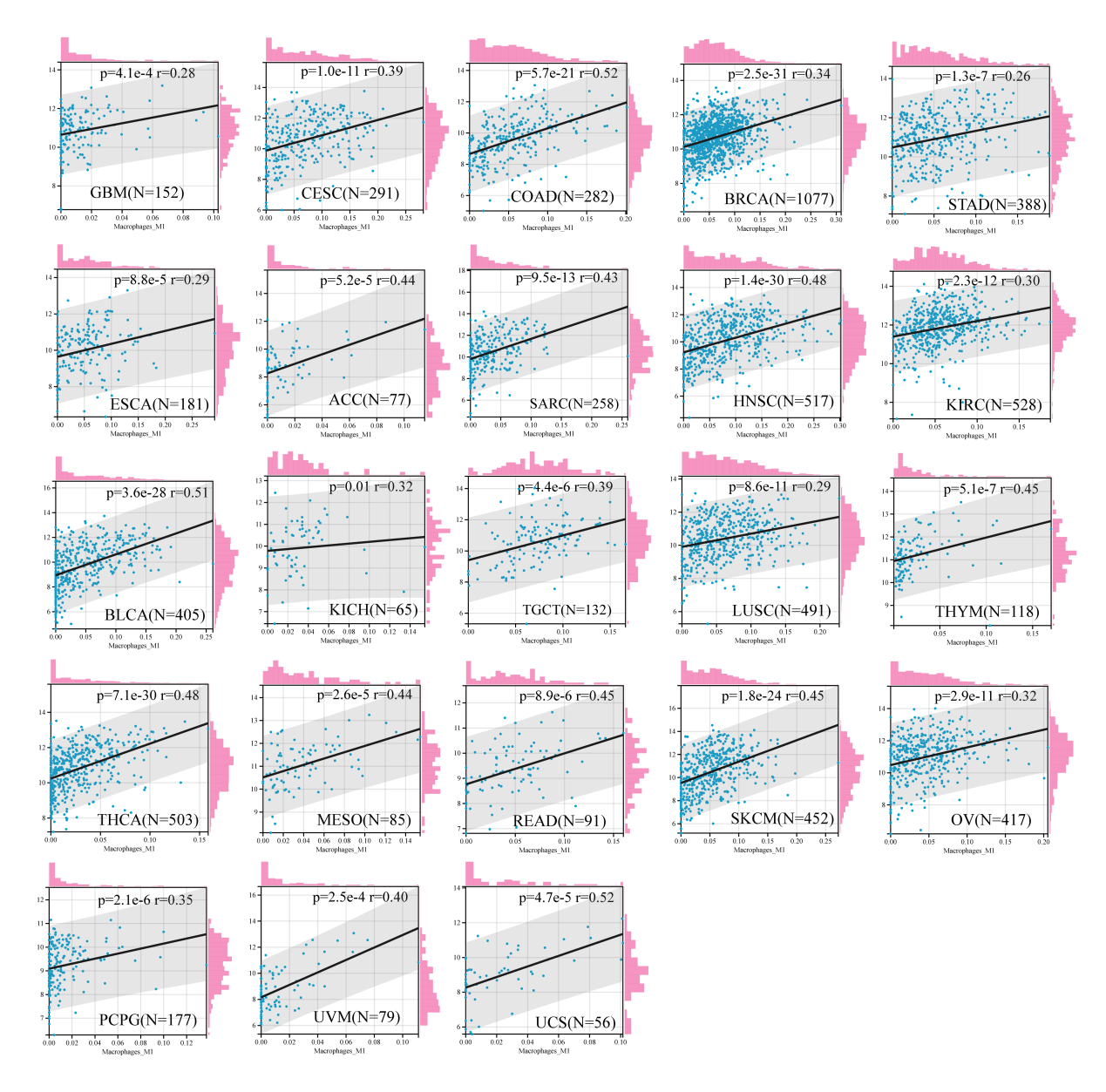


**Supplementary Figure 2. Correlation of CD74 Expression with M1 Macrophages.The results showed that CD74 expression in most tumors was significantly and positively correlated with M1 Macrophages.**


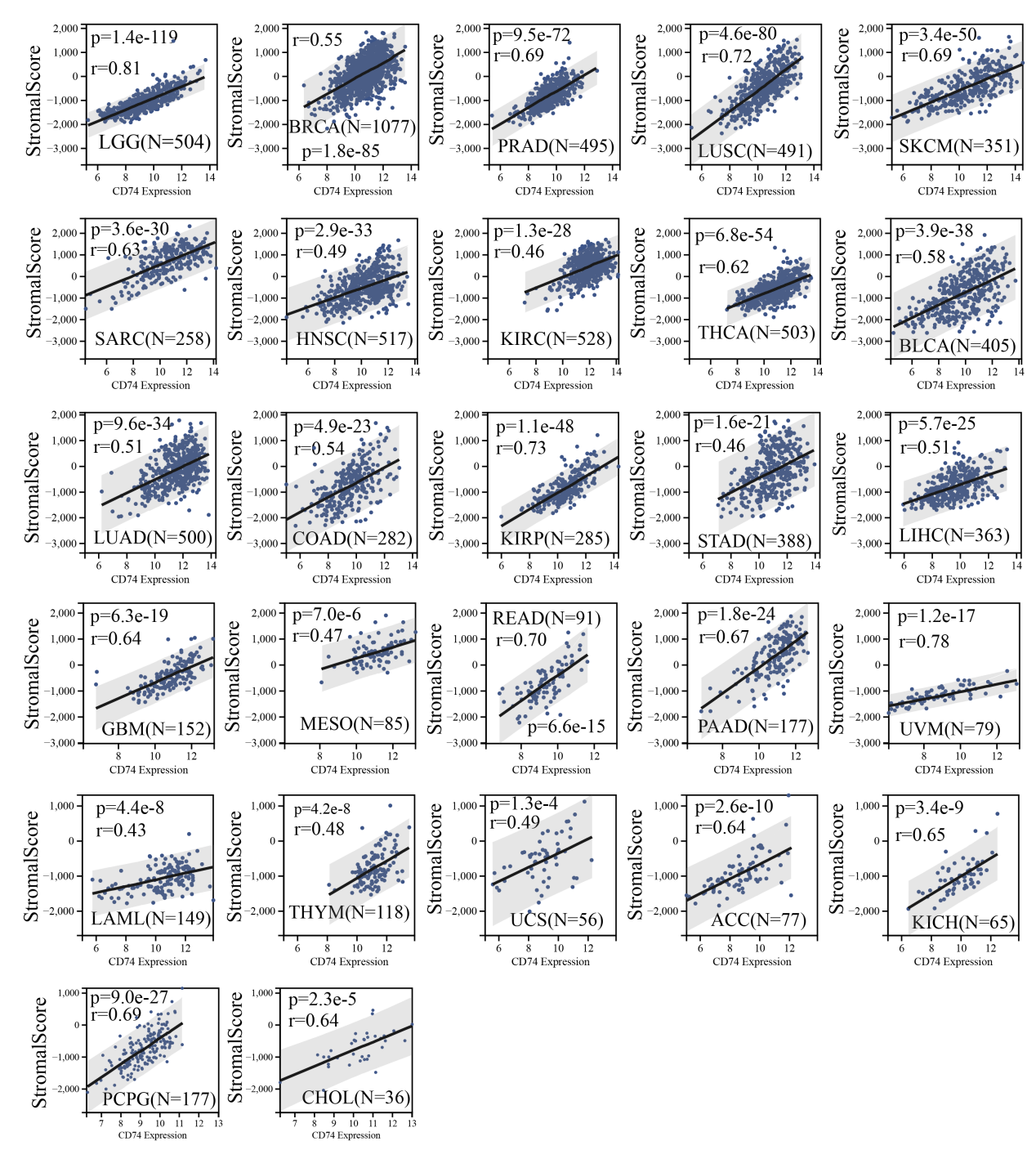


**Supplementary Figure 3. The relationship between CD74 expression and** **StromalScore.The results showed that CD74 expression in most tumors was significantly and positively correlated with StromalScore.**

**
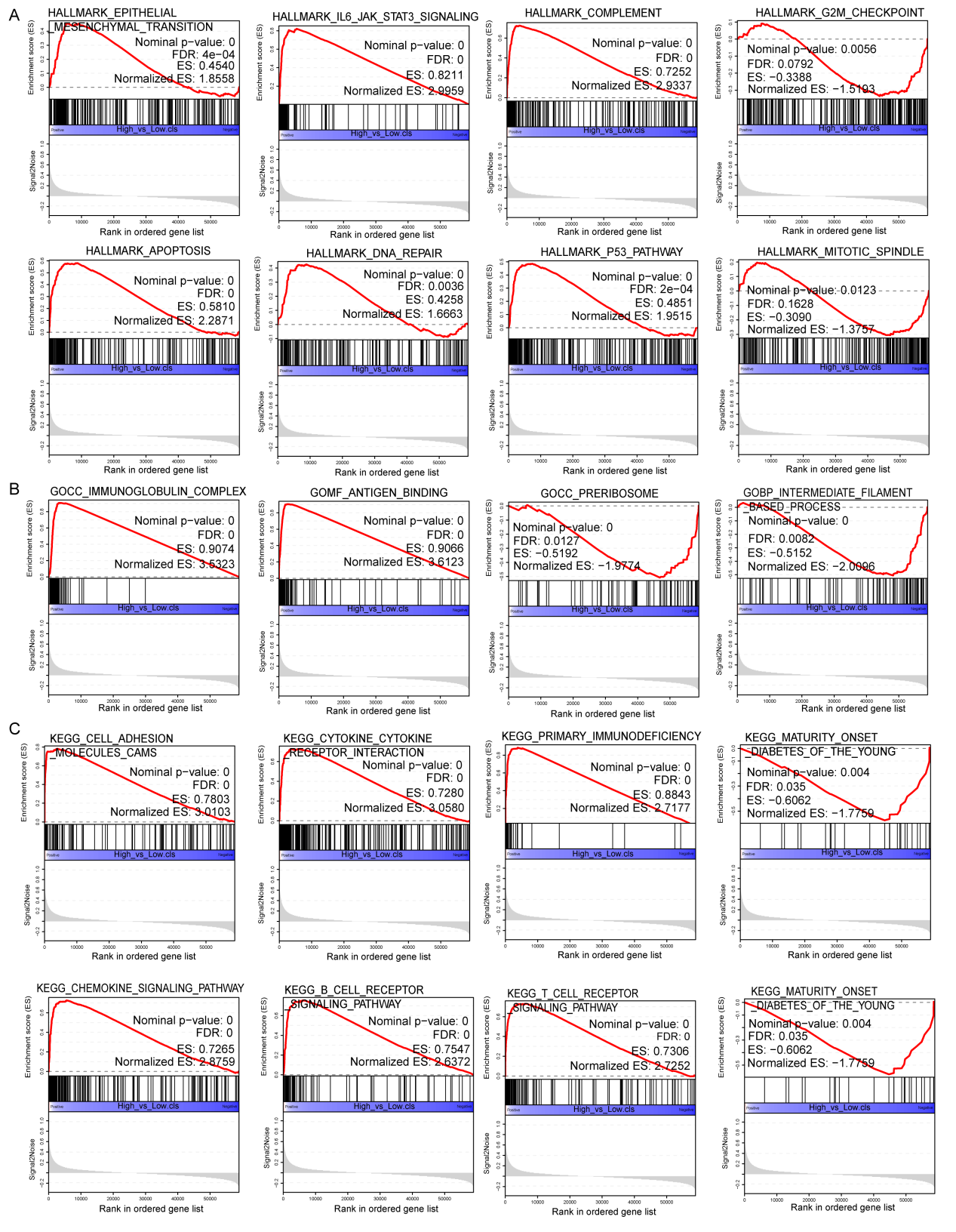
**

**Supplementary Figure 4. Analysis results of GSEA. (A) HALLMARK analysis of CD74 in STAD. (B) GO functional annotation of CD74 in STAD. (C) KEGG pathway analysis of CD74 in STAD. The chart above shows only some of the results. Peaks on the upward curve indicate positive regulation and peaks on the downward curve indicate negative regulation.**
